# Relationships between Recreation and Pollution When Striving for Wellbeing in Blue Spaces

**DOI:** 10.3390/ijerph19074170

**Published:** 2022-03-31

**Authors:** Clifton Evers, Cassandra Phoenix

**Affiliations:** 1 School of Arts and Cultures, Newcastle University, Newcastle NE1 7RU, UK; 2 Department of Sport and Exercise Sciences, Durham University, Durham DH1 3LE, UK; cassandra.phoenix@durham.ac.uk

**Keywords:** blue space, pollution, wellbeing, sport, leisure, surfing, recreation, environment, water

## Abstract

Our aim for this research was to identify and examine how recreation enthusiasts cope with and mitigate the violence of pollution as they strive for wellbeing in polluted “blue spaces” (e.g., seas, oceans). Our methodology to undertake the research was ethnography (online and offline), including autoethnography and informal interviews (40). The study proceeded from a constructivist epistemology which emphasizes that knowledge is situated and perspectival. The study site was a post-industrial area of northeast England where a long-standing but also rapidly growing surfing culture has to live with pollution (legacy and ongoing). We found evidence of what have become quotidian tactics that attach to themes of familiarity, embodiment, resignation, denial, and affect/emotion used by enthusiasts to cope with and mitigate the violence of pollution. We argue that by necessity some surfers are persisting in striving for wellbeing not simply *in spite of* pollution but rather *with* pollution. We assert surfers enact a “resigned activism” that influences their persistence. We extend critical scholarship concerning relationships between recreation, blue spaces, and wellbeing by moving beyond a restrictive binary of focusing on either threats and risks *or* opportunities and benefits of blue space to health and wellbeing, instead showing how striving for wellbeing through recreation in the presence of pollution provides evidence of how such efforts are more negotiated, fluid, situated, uncertain, dissonant, and even political than any such binary structure allows for.

## 1. Introduction

Health research connecting with aquatic environments, has a long history of focusing almost exclusively on the threat that these “blue spaces” (e.g., rivers, seas, lakes, and canals) can pose to human health and wellbeing. Drowning is a common cause of unintentional death [1]. Poor water quality causes respiratory, gastrointestinal, eye, ear infections as well as leading to and exacerbating water-borne diseases [2,3].

However, in more recent years, the focus on threats has been met by a growing body of multi and interdisciplinary research committed to investigating the opportunities that blue space holds for enhancing health and wellbeing [4,5,6,7]. Recreational activities in blue spaces are said to enable embodied sensual pleasures [8,9]; heightened spirituality [10]; creativity and resilience [11]; positive affective, imaginative, and emotional experiences [12,13]; respite from suffering [14,15]; mindfulness, connection, contentment, life-affirmation [16,17]; and problem-solving [18]. Moreover, recreational activities in blue space are argued to provoke pro-environmental holistic connections with the wider world (human and non-human) that link the health of marine environments and people [19,20,21]. As evidence of wellbeing experienced by spending time in blue spaces has grown, it is perhaps unsurprising that aquatic environments such as the coast have become increasingly framed within health research, policy, and practice as a viable public health resource [22,23,24]. 

The opportunistic framing of blue spaces and associated outdoor recreation has not passed without criticism [25]. An overemphasis on the positive wellbeing or health-enabling dimensions of blue space does not adequately acknowledge how aquatic places are also sites of exclusion and oppression informed by, for example, racism [26,27,28], sexism [29,30,31], violent territorialism [32], and colonialism [33,34]. Further, much of the opportunistic-led literature has privileged Eurocentric discourses that have not adequately situated knowledge and experiences of relationships between wellbeing, recreation, and blue spaces [35,36,37]. We locate our own contribution in a critical approach to blue spaces because it prompts more reflexive, perspectival, and situated lessons about how relationships between recreation, pollution, and wellbeing happen in blue spaces. This critical approach does not smooth over the complexities, nuances, similarities, contradictions, and paradoxes that people experience when “living with” pollution to make generalizations. Instead, it promotes understanding of the multiple lively realities and unique barriers that shape how people strive for and indeed experience wellbeing during recreation in polluted blue spaces. 

In this article, we extend the body of critical blue space scholarship by analyzing the seemingly contradictory practice of a case study aquatic recreation group—surfers—seeking wellbeing and “stoke” in contaminated waters. Stoke is a fully embodied sense of satisfaction and feeling good that informs surfer perceptions of wellbeing. We ask: how do surfers cope with and mitigate pollution when striving for wellbeing and stoke in blue spaces? Our emphasis is on the quotidian tactics for living with pollution when striving for wellbeing, rather than on the what or why of pollution or the surfers’ perspectives of wellbeing. We extend critical blue space scholarship by moving beyond the potentially restrictive binary of focusing on either the threats and risks *or* opportunities and benefits that recreation in contaminated aquatic environments can present. 

There is a long history of considering connections between recreation (or the synonym: leisure) and wellbeing [38,39]. The term “wellbeing” is not always explicitly used in respective literature, but relatable ones are such as therapeutic, happiness, life satisfaction, a sense of meaning, quality of life, personal growth, and self-care [40,41,42,43,44,45]. Louise Mansfield, Norma Daykin, and Tess Kay explain how “multiple and contested meanings” of wellbeing are used to “articulate the complex socio-cultural, personal, political and policy relevance of leisure for wellbeing” [38] (p. 1). Some researchers focus on personal individual measures and perceptions of meaningfulness, autonomy, mastery, capabilities, belonging, and satisfaction in relation to “subjective wellbeing” [46,47]. Others emphasize social factors such as financial security, social freedoms, and social bonding [48,49]. We use the concept wellbeing in a way that resonates with more recent conceptualizing typically from within human geography literature, where it is viewed as the lively, situated, and relational qualitative evaluation of whether capabilities (what can be or happen) are enriched or not in ways that that cut across the personal, social, and material world [50,51,52]. 

Personal, social, cultural, and material relationships between surfers and pollution are longstanding. Globally, surfers have a long history of having to live with pollution when pursuing their recreation in blue spaces. For instance, during the 1970s and 1980s at Bondi Beach in Sydney, Australia a near-shore sewage outfall spewed condoms, chemicals, and the famous “bondi cigar” (human faeces). Surfing despite the pollution, and even having the “proud experience” of bumping into a “bondi cigar”, contributed to identity-formation [53]. Moreover, surfers are high-frequency year-round users of blue space for recreation; they immerse themselves in the waters for long periods (several hours) so exposure and familiarity with pollution is extensive, and they frequently ingest polluted water when surfing so commonly experience illness [54,55,56,57]. As Clifton Evers argues, surfers “think, feel, and act with the rhythms, flows, surges, and throbbing of oceans *and pollution*” [58] (p. 423). The history of Bondi surfers constitutes one of many examples showing how surfers develop intimate relationships with ecologies [20,35,59,60]. It is argued in popular media and academic literature that through their encounters with blue spaces and learned “ecological sensibilities” surfers experience wellbeing (physical/psychological), health, healing, belonging, environmentalist awakening attitudes, and spirituality [14,16,20,61,62]. Pollution is part of any ecological sensibility [58,63]. 

All blue spaces are polluted. Pollutants include sewage, chemicals, agricultural and urban runoff, dumping of industrial waste, plastics, and radioactive substances [64]. In 2019, *The Times* newspaper reported that “dangerous pollutants in England’s waterways have reached their highest level since modern testing began” [65]. In 2020, water companies discharged raw sewage into waterways in England more than 400,000 times [66]. Given the high pollution levels, people become sick during their aquatic recreation [67] and some cease participation [68], while others continue [58]. Our focus in this article is on those who continue participation. 

## 2. Methodology

Our case study is surfers in northeast England. We learned from surfers who live along a coastline stretching from the “working” River Tyne in county Tyne and Wear to the seaside area of the conurbation of Teesside. The UK’s largest chemical complex remains located in Teesside, which for over two-hundred years was a key industrial hub of England—petrochemical, mining, agricultural, manufacturing, energy, demolition, shipping, and shipbuilding [69,70]. The coastline has experienced long-term deindustrialization. Large areas of the coastline are damaged due to pollution (legacy and ongoing). Pollution, de-industrialization, and lack of investment have negatively affected the broader local communities in terms of their life opportunities and capabilities. Unemployment levels are high. People in this coastal region seek out wellbeing through their blue space recreation. While the smells, sounds, sights, cultures, and stories of blue space recreation along this coastline may lead to a subjective sense of wellbeing, striving for wellbeing requires negotiating with smokestacks, winces, sewage, and illness. Exemplifying how local surfers live materially and culturally with pollution when pursuing their recreational activity, it is noteworthy that several surf-breaks along this stretch of coastline have been named after their perceived sources of contamination: “Oilies”, “Shitpipe”, “Wastelands”. 

This paper draws from ongoing international research into surfing scenes led by Clifton Evers that has spanned 22 years, focused most recently on the United Kingdom-based northeast surf scene and evolving into a more collaborative venture in 2019 when joined by Cassandra Phoenix. The study proceeds with the premise of a constructionist epistemology that emphasizes how knowledge is situated and perspectival as well as that data are constructed during the activity of the research process rather than found “out there” [71]. 

We report on data constructed through an ethnography (2016—ongoing), including forty informal interviews to date. The term “informal interviews” has a number of other synonyms such as “informal conversations”, “unstructured interviewing”, and “ethnographic interviewing” [72]. These interactions are a constituent part of participant observation, which is the main method ethnographers use to construct data and meaning with research participants. An informal approach to interviews is what the surfers felt most comfortable with. We had conversations with surfers in car parks, walking together, and surfing together [16,73,74]; during participation in a 3-day women’s only surf camp and women only surf lessons; and when attending film nights, beach cleans and other events organized by the local surf community. When meeting participants we elicited discussion about the research question by using the prompt word “pollution”. Some enthusiasts picked up on this prompt while others did not. It was a light touch approach to prompting and participant-led conversation and reflection. Conversations were reflexively written up as ethnographic field notes, but also sometimes audio-recorded following permission from participants and signing of appropriate ethics forms. We regularly had audio-recorders with us. We also constructed data and meaning through autoethnography, which is used by some researchers who are active in the site and cohort they are studying [75]. It is an approach to qualitative research which sees the researcher and what they learn in situ as socially, materially, and culturally accountable parts of constructing data, analysis, and findings. The ethnography involved online research as enthusiast communities bridged offline/offline settings. We were permitted to participate in online community through which we were able to provide further context for analysis, build rapport with participants, note tactics and opinions, conduct some informal interviews, and generate themes [76]. We paid attention to comments, questions, memories, recommendations, and images about pollution that were posted by users across multiple social media platforms (Twitter, Facebook, Instagram, WhatsApp). We selected these platforms because they were the ones favored by the enthusiasts. In doing so, we contribute to a growing body of digital qualitative research in sport and physical activity [76,77,78] including surfing [79,80,81]. We used reflexive thematic analysis to analyze the data [82]. Reflexive thematic analysis generates themes via a recursive and inductive processing of research aims/questions, field, data, coding, theory, and researcher subjectivities. 

Our biographies directly affected our research. We followed Rebecca Olive and Holly Thorpe’s reflexive collaborative approach to doing research [83]. To that end, prior to discussing our research findings, it is appropriate that we acknowledge our own (blue space) biographies. Cassandra is a beginner surfer who identifies as a white British woman and comes from a middle-class background. Clifton meanwhile, has surfed all their life, is an immigrant, and identifies as a white male coming from a working-class background. Our different levels of skill and experience means that we move in disparate circles within the local surf community and that certain aspects of surfing are important to us in different ways (e.g., quality and size of waves). It is also important to situate the knowledge. The northeast of England has the highest percentage of the population from the white British ethnic group. The surfing population we met along this coastline was overwhelmingly white. What we learned and share in this paper occurred through this perspectival lens. There was class diversity in terms of the surfers we met during the ethnography, some surfers being from wealthy areas and others from working class. Race and gender as well as surfing ability shaped our access to certain surf-breaks and when each of us could do fieldwork. For example, many of the most polluted zones, were also the most isolated and risky. These were frequented overwhelmingly by experienced male surfers, with Clifton having privileged access. His and the respective cohorts’ exposure to pollution was, therefore, more frequent and more intense. He had access to and engaged with experienced enthusiasts with long histories of living with pollution. Experienced surfers can be distrustful and judgmental of newcomers [84]. Trust and judgement, however, work both ways, with newcomer and intermediate surfers feeling equally inhibited to sound things out or be seen to ask “silly questions” in the presence of those deemed expert. Furthermore, while still the minority, women’s surfing has experienced unprecedented growth in recent years [29,85]. This is mirrored on the northeast coast of England, where a burgeoning women’s surf scene had been slowly growing pre-2018 but has rapidly grown since. Cassandra’s position as a relative newcomer (both to the surf scene and to the northeast coast of England) proved advantageous for connecting to women who surf in addition to those new to surf culture. Our data and assertions reflect the above epistemic standpoint and that should be borne in mind by the reader. Despite being aware of alternative standpoints and further diversity of experiences, analysis of the demographic differences shaping the coping with and mitigating of pollution when striving for wellbeing during recreation in polluted blues spaces is beyond the scope of this article. 

Theoretically, we drew on the work of Ajiang Chen, Pengli, Chen, and Yajuan Luo [86]. Through their studies of “cancer villages” in China they explain that practical acts of daily resistance and adaptation to pollution highlights how politically marginalized, less formally educated, and poor residents who often rely (or have relied) on polluting industries, adapt to live with polluting industries and damaging government policy. Engaging in “ordinary” cultural practices—e.g., not washing in and drinking from local rivers, wearing masks, avoiding local foods—lessened the purchase industry, government, and associated pollution had on their life over time. For example, their health and wellbeing. Anthropologist Anna Lora-Wainwright’s fieldwork was conducted alongside Chen et al. and further contributed to our understanding of this area or research with her concept “resigned activism” [87]. The concept refers to how residents respond to pollution not only through conventional forms of activism such as protesting, lobbying, and lawsuits but also in ways that would not normally be identified as activist at all. For example, the “ordinary” daily practices noted by Ajiang Chen, Pengli, Chen, and Yajuan Luo.

Additionally, what is also helpful for understanding and appreciating what paying attention to ordinary daily acts people undertake to live with pollution can teach us is Michel de Certeau’s theory about how practices of everyday life are political [88]. De Certeau argues for the significance of grasping the logic of individual ordinary practices that in turn allow us to understand the bigger picture. Practices are connected, and a network of practices can constitute, knowingly and unknowingly, political activism. De Certeau explains how political activism can proceed through strategies and tactics. Strategies operate from a position of access to institutions, regulations, and discourses that rationally construct and demarcate a space of power by establishing and validating what “is orderly”, “reasonable”, and “knowable” in that space (see cities, organizations, states, non-governmental organizations). Conventional forms of activism are strategic in that they involve negotiating with and through established institutions, regulations, and discourses thereby legitimizing them. In contrast to strategies, de Certeau explains how tactics can be considered as creative instances of poaching, interfering, subverting, transforming, eroding, and evading strategies. Tactics are used by those without power to identify and act on opportunities—however fleeting—to contest, mitigate, cope with, and evade the strategies that favor and legitimate the powerful. Tactics involve using what is at hand and are ways those without power may struggle in a piecemeal way against and indeed survive the strategies that are doing violence to them. 

## 3. Findings and Discussion

Many long-term northeast surfers developed a sense of self in relation to heavy industry and pollution. A theme of “familiarity” was constructed through discussion and fieldwork. As elders told us through informal interviews:

I learned to surf in poo in the 1980s

(informal offline interview)

The air pollution from the chemical plant, the muck from the old steelworks, the sewage, and the mine runoff are normal … it’s an industrial area, what do you expect?

(informal offline interview)

Lora-Wainwright calls such a familiarization or naturalization process “toxic nature”, whereby pollution becomes an all-pervading part of the natural environment affecting its appearance, how it functions, as well as how inhabitants look, feel, act, and know [87] (pp. 11–14). The pollution can arrive spectacularly, such as via oil spills or sewage overflows after heavy rain. However, pollution can also be “slow violence”. Robert Nixon explains slow violence “as a violence that occurs gradually and out of sight, a violence of delayed destruction that is dispersed across time and space, an attritional violence that is typically not viewed as violence at all” [89] (p. 2). The causes of the slow violence—including strategies of capitalism, resource extraction, colonization, classism—can become veiled, disguised, and elusive if they slowly unfold or principally happen ”over there”. There is an associated gradual erosion of ecological relationships and associated life capabilities, health, and wellbeing. Yet, as the comments demonstrate, while the pollution has become familiar and natural it is not out of sight or “over there” in any way. Geographer Thom Davies challenges Nixon by pointing out that slow violence is never “out of sight” or “over there” for everyone [90]. By way of an empirical case study of a polluted region in southern Louisiana, USA (nicknamed “Cancer Alley”) Davies shows how the everyday exposure that is living “in the midst of toxic geographies” (p. 2) means that while Nixon’s concept of ‘slow violence’ is predicated on being “spectacle deficient” that for many communities there is a highly visible “tangible brutality” (p. 13). The violence is “here and now” and “plain to see” (p. 13).

The surfers’ bodies are in communion with the violence of pollution. Embodiment was another theme we constructed through our analysis. There were examples of this communion and embodiment in online conversations:

From when I started SUPing [stand up paddleboarding] a few months ago I’ve had a problem with my nose and ear, after 3 courses of antibiotics gp has referred me to an ENT [Ear Nose Throat] specialist—wondering if this has anything to do with the quality at [name] beach [shrugging ‘dunno’ emoji].

[women-led SUP WhatsApp group]

-------

A: Girls… just put my wetsuit on and it was a bit damp. It stinks. However, that’s another issue. My legs are like pins and needles. They’re so itchy, does anyone else know what this is? Heading out soon.

B: Not sure if it’s the same but sometimes (usually after being in A LOT, usually (place) or (place), or after a period of heavy rain…) I can come up in welts in areas where the water would “sit” (wrists, hips etc.). Clearly some sort of allergic reaction or reaction to something nasty. Benadryl and Milton [anti-histamine] /Piss Off is your friend [antimicrobial wetsuit cleaner] and full fat coke [Coca Cola] for your insides too.

These online observations were supported by comments made in the surf and post-surf during a swell that arrived after heavy rainfall at a notoriously polluted surfing location at the mouth of a river. The rainfall led to sewage overflows as well as high levels of industrial and agricultural runoff.

[Name] was puking his guts up while paddling out. (informal offline interview)

I’ve been sick for days after [surfing]. Middens [a surf break] is terrible for that. Earaches. Infected eyes. Stomach bugs. I’ve had it all. (informal offline interview)

Pollution shapes the surfers’ experiences of their bodies. They told stories of illnesses including gastrointestinal, ear infections, sore throats, coughs, diarrhea, fever, and vomiting. Similarly, during his lifetime of surfing, author A has had all these illness and infection experiences, including methicillin-resistant staphylococcus aurus infections (MRSA). MRSA is a by-product of the agricultural use of antibiotics given to livestock during industrial farming and excreted in urine and faeces, then dispersed through groundwater and surface runoff. The illnesses cause headaches, muscle pain, fever, cough, chest pain, shortness of breath, and rashes. It is unavoidable that pollution enters the bloodstream, cells, and brain. Writing in the environmental humanities, Stacy Alaimo argues “all creatures as embodied beings, are intermeshed with the dynamic, material world, which crosses through them, transforms them, and is transformed by them” [91] (p. 435). In light of Alaimo’s point consider the following online exchange:

[Beginner surfer]: Hi! I’m wondering if people still head into the sea when there are pollution warnings? Thanks.

[Experienced surfer]: At this point, I’m part pollution part human, so yes, I do, haha.

[6 separate emoji responses of laughing faces]

Many of the surfers including Author A continue to surf if the waves are excellent quality even if the water is heavily polluted. They are resigned to the violence of pollution. Resignation was another theme arrived at. We assert that the resignation is a tactic to cope with the ever-present pollution so that the surfers can continue to strive for wellbeing and stoke. The resignation signals are striving with pollution for wellbeing that in turn prompts the surfers to identify and come up with tactics to cope with and mitigate pollution. For example, there is an urban legend that drinking full fat coke will clean your gut after surfing. Some surfers carry a bottle of fresh water and soap to wash off if surfing after rain. The washing practice has become ritualized due to repetition.

When family and friends question the risk, say, Author A takes he plays down or even denies the effects of the pollution. Denial was another theme generated through the research process. The denial is also a tactic; that is, a psychological defense against the violence. If Author A thinks about the pollution too much his striving for wellbeing is curtailed. Other surfers also employ the denialism. Dwelling on the threats and harms would interrupt the stoke and wellbeing they seek through surfing. A tactics that is employed in relation to the denialism involved questioning and undermining correlations between recreation, pollution, and health to allay concerns. While some surfers get sick others do not. And none of the surfers have undergone specific medical testing to evaluate correlations. Diseases and conditions that could be attributed to surfing in polluted waters are contested. Some of the surfers challenged official discourses about the health effects of pollution, particularly when those discourses would interrupt their striving for wellbeing and stoke through their highly valued recreation. 

**Figure 1:**
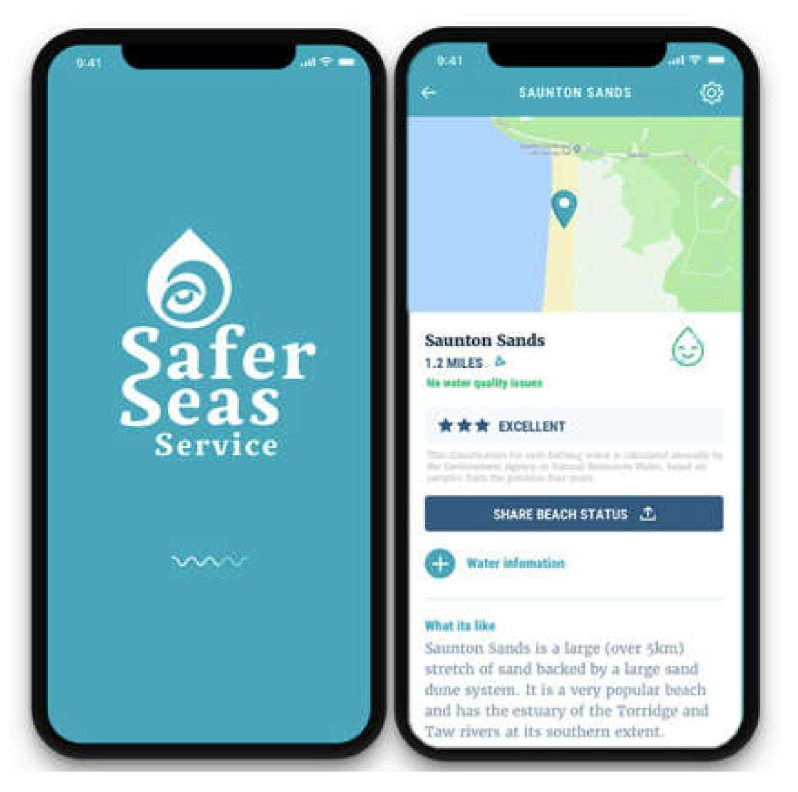
Screenshot of the Safer Seas Service app.

Some of the surfers paid attention to official cautions. Beaches at the study sites have Environmental Agency warning signs and there are online notifications. The “Safer Seas and Rivers” app (Figure 1), developed by UK surfer-led environmental activist NGO “Surfers Against Sewage”, is a well-established resource within the UK surf scene. It is an example of how some surfers organize their environmental concerns around activism [92]. 

This App is free and monitors the water quality at over 400 locations around UK rivers and coastlines, providing warning of pollution incidents in real-time. The App helps with deciding where to surf (or not surf), particularly following periods of heavy rain. The App data is sourced from the UK Environmental Agency and supplemented by members of the community; a feature welcomed for its acknowledgement of “expertise by experience” alongside objectively driven data alerts. One participant claimed

The app is good and you can also use it to log if you see poor water quality when there’s no alert!

[thumbs up emoji] [women-led WhatsApp group]

However, apps such as Safe Seas were not consumed or adhered to without question. One surfer told Author A via an online Facebook message, “I don’t need an expert to tell me about pollution because I already know. I grew up with it.” Deference to environmental experts and institutions (e.g., Surfers Against Sewage, the Environment Agency) relative to environmental risk may be expected but some of the surfers preferred to prioritize local histories or valuations of risk based on theirs and peers familiar embodied knowledge of pollution and past effects (or not), resignation, and denialism in relation to the impact on their striving for wellbeing and stoke. As Lora-Wainwright explains, there are diverse languages of valuation that come about as “what has become considered as healthy—or at least a bearable environment in the context of toxic natures” [93] (xxix). 

Surfers develop a situated, relational, and sensual polluted “surfer’s gaze”. A surfer’s gaze is formed through a tying together of cultural discourses with the minutiae of weather and coastal contingencies [87]. For example, fast moving water signaling the rips, whitecaps indicating strength of wind, bubbles from a reef indicating depth of water. The gaze is a key part of a surfer’s ecological sensibilities. Clifton Evers extended understanding of the surfer’s gaze to include sound, smell, taste, and touch [94]. Evers went further again to note how toxic nature extends the surfer gaze to the registering, perceptions, and knowledge of pollution [73]. An exchange Author A had with a local surfing elder brings into sharp relief the polluted surfer gaze:

See. There. Wait for it. There! That orange water bubbling up. There’s a pipe. I’ve seen it at low tide. My dad told me about it too, it’s been there since he was a child. It’s freaky, right? … Surf the south end (of the beach) when you see that.

(informal offline interview)

The surfer’s gaze is a tactic to cope with and mitigate pollution. However, not all pollution is anthropogenically “sense-able”. For example, in Japan surfers used personal Geiger counters following a magnitude 9 earthquake and tsunami in 2011 disabled the Tokyo Electric Power Company’s (TEPCO) Fukushima Daiichi Nuclear Power Plant causing a nuclear accident. Given this, we assert that pollution evidences how technology is not separate to but imbricated and inseparable from human capabilities and senses and in turn the surfer gaze and ecological sensibilities [58,95]. Technology is now part of the tactics for striving for wellbeing in polluted blue spaces. 

Underpinning the surfer sensibilities and concomitant tactics for coping with and mitigating pollution is an affective and emotional life. Pollution is emotional and affective, a further theme that was constructed with participants. What do we mean by affect and emotion? There are many interpretations of affect, some prioritizing anthropocentric somatic sensations of forces-institutional, political, cultural, spatial, technological, and material—expressed through bodies and others a more abstract distributed more-than-human agency and expression. Our focus here is on the former [96]. Expression may occur as facial expressions (e.g., an impromptu smile), pitch of voice (e.g., startled cry), gestures (including the restriction of such), quickened beat of the heart, clammy hands, a shiver running across the skin, and a struggle to breathe. The somatic sensations and expressions co-assemble with individual biographical experiences and memories. Consequently, there occurs individuation, explanation, manipulation, communication, labelling, and organizing of affective activity for meaning making. The meaning making is what we are calling “emotion”, i.e., semiotic explanation of pride as proud, anxiety as anxious, fear as afraid, anger as angry, and so on. Mind you, there is no simple affect (physical/emotion) and semiotic division but a contingent, constructive, and flexible feedback loop [97] (p. 19–20). As Klaus Scherer explains, affects involve the “synchronous recruitment of somatic and mental resources” [98] (p. 314). The affect-emotion process can be seen in the following fieldnote about by Author A about a toxic communion experience:

I paddle through patches of rainbow-colored foam that clings to my wetsuit and face. Oil not only clings to bodies as foam but in the form of neoprene (a processed petrochemical rubber used to make wetsuits). The joy of going surfing mutates into disgust. I try to brush the foam off, but it is sticky. The disgust mutates to anger. Who or what is to blame for this pollution? The owners of the now closed mine that continues to leech lead and mercury into the nearby river? The chemical plant? Agricultural industry runoff? Most likely it is the water company that manages the Combined Sewer System at this beach. My muscles tense.

[Author A]

Author A tends to feel angry at “them”—the companies and government. Anger constructs an object to be angry about. The anger is entangled with ascribing guilt and is outward looking. Other surfers also expressed anger about “them” during the fieldwork. Mind you, there is an environmentalist “cultural dissonance” given surfers continue to favor equipment manufactured from petrochemicals and few undertake environmental activism despite a semiotic history that would suggest otherwise [99,100,101]. When Author A expressed his anger to another elder local surfer the latter shrugged to indicate a sense of powerlessness and responded

What are you going to do? … Protest? … What good is it calling the media? … I’ve done that and here we are … The government has said how many times they’d spend the money to clean it [soil and water in the area] up?

(Informal offline interview)

A history of deindustrialization interpreted as an abandonment by government and industry and a long list of previous failed efforts to clean the blue spaces up has led to a distrust of strategy and fuels resignation. The resignation can feel shameful, even though the violence is not personally one’s fault. As environmental sociologist Blanche Verlie writes in regard to climate change, “we can and do feel violences inflicted on the atmosphere and broader planetary relations in our own bodies as these violences are also inflicted, in some ways, on ourselves” [102] (p. 7). One surfer noted:

I know it not necessarily *my* fault, even though I worked in the steelworks [now closed] and I don’t do much environmentalism stuff … I know it’s a big problem. I sometimes feel a bit ashamed about what we did at work … dumping all sorts of stuff into the river … so I’ll help out SAS [Surfers Against Sew age] but I lose enthusiasm … it can get depressing living here … I like going for a surf to wash off my worries but the pollution is still there.

[our italics] (informal offline interview)

Cultural theorist Elspeth Probyn makes the argument that while shame provokes discomfort, distress, and disappointment it can also be a productive force [103]. Shame is bound up with an interest and investment in relationships (material and social) as well as caring about these so as to improve their quality. We have to care about something or someone to feel ashamed. Shame may become a vehicle to investigate one’s actions, history and mistakes. In short, to care. In addition to shame, we assert anxiety was evident during the above conversation. The anxiety being fueled by the knowledge that people employed in the local industries such as the steel industry, demolishing of oil rigs, and chemical plant need to put food on the table. Pollution remains tied to livelihoods in this area. As Lora Wainwright explains, “pollution cannot be separated from the many other challenges locals face—such as finding work, paying for healthcare, and improving their family homes” [104] (13–14). Or as miners explained to anthropologist June Nash during her study of Bolivia mining, “We eat the mines, the mines eat us” [87] (p. 1). The shame and anxiety mingle with the striving for wellbeing and stoke through surfing. Riding contaminated waves can feel joyful and exciting for surfers, rejuvenate them despite the violence being geared towards them. This is evidence of shifting alliances when striving for wellbeing in relational blue spaces. Sometimes the affects and emotions can at times be tactical given that may afford ”a moment in time” to care and even seek out hope, but strategic process (e.g., capitalism) apply pressure to those moments and work to shut them down. In turn, we assert that this is evidence of how pollution complicates any binary view of striving for wellbeing and stoke through blue space encounters that would focus on either the threats and risks *or* opportunities and benefits.

## 4. Conclusions

This article extends critical blue space scholarship by attending to the seemingly contradictory practice of surfers who float, paddle, glide, and submerge themselves within contaminated waters in the pursuit of wellbeing or stoke. The resigned activism of the surfers troubles a common binary structuring of blue spaces that have typically focused on either the threats to *or* benefits for health and wellbeing that recreation in and around aquatic environments can present. As a starting point on a “polluted leisure” phase of critical blue space research, through our ethnography, we have shown how some surfers tactically strive for wellbeing not *in spite of* pollution, but rather *with* pollution. 

Through our research we identified a resigned activism occurring as quotidian tactics attached to themes of familiarity, embodiment, denial, resignation, and emotion/affect. We also asserted that the surfers’ tactics arguably function as a mode of resistance to strategic arrangements of society that do violence against them through pollution, including challenging the hegemonic strategic knowledge and expertise that produced the conditions they are forced to live with and that would serve to diminish their capacity for striving for wellbeing and stoke. From here, research would do well to shift away from an exclusive focus on what being in blue space does *to* or *for* us, and instead further incorporate an attentiveness to how we live *with* pollution in blue spaces as part of a wider situated, relational, and dissonant wellbeing project on a permanently polluted planet. Recent revelations of the scale at which pollution is regularly discharged into the sea (at least around UK shorelines), alongside sharp increases in the number of people taking to the water (coastal and inland) for recreation demonstrates the timeliness of this work. There is much work to be done. Future research could make valuable contributions to this knowledge by focusing explicitly on some of the dimensions signaled here, but beyond the capability of a single article to interrogate fully. For example, there is a need to explore racialized, gendered, and indigenous/first nation experiences and perspectival (to name a few) relationships with “polluted leisure” in blue spaces. We might also examine the process by which newcomer surfers *learn* resigned activism (or otherwise), including the tactics to deploy. Finally, our interest in the post-industrial northeast of England was significant given its lasting impact on the local environment, along with place-based identities for many of the communities along that stretch of the coast. Blue spaces are relational, and accordingly, further investigation across various geographies is warranted. We hope that our work in this area acts as a useful point of departure. 

## Data Availability

The data presented in this study are available on request from the corresponding author. The data are not publicly available due to privacy.
